# Detecting Overlapping Communities in Modularity Optimization by Reweighting Vertices

**DOI:** 10.3390/e22080819

**Published:** 2020-07-27

**Authors:** Chen-Kun Tsung, Hann-Jang Ho, Chien-Yu Chen, Tien-Wei Chang, Sing-Ling Lee

**Affiliations:** 1 Department of Computer Science and Information Engineering, National Chin-Yi University of Technology, Taichung 41170, Taiwan; 2 Department of Applied Digital Media, WuFeng University, Chiayi County 62153, Taiwan; hhj@wfu.edu.tw; 3 Department of Computer Science and Information Engineering, National Chung Cheng University, Chiayi 62102, Taiwan; kw.15@hotmail.com (C.-Y.C.); felicitylab@gmail.com (T.-W.C.); singling@ccu.edu.tw (S.-L.L.)

**Keywords:** data mining, community detection, overlapping communities, modularity

## Abstract

On the purpose of detecting communities, many algorithms have been proposed for the disjointed community sets. The major challenge of detecting communities from the real-world problems is to determine the overlapped communities. The overlapped vertices belong to some communities, so it is difficult to be detected using the modularity maximization approach. The major problem is that the overlapping structure barely be found by maximizing the fuzzy modularity function. In this paper, we firstly introduce a node weight allocation problem to formulate the overlapping property in the community detection. We propose an extension of modularity, which is a better measure for overlapping communities based on reweighting nodes, to design the proposed algorithm. We use the genetic algorithm for solving the node weight allocation problem and detecting the overlapping communities. To fit the properties of various instances, we introduce three refinement strategies to increase the solution quality. In the experiments, the proposed method is applied on both synthetic and real networks, and the results show that the proposed solution can detect the nontrivial valuable overlapping nodes which might be ignored by other algorithms.

## 1. Introduction

Determining the group with some particular properties helps the analysts to capture the common properties from the members in the community. Many applications could be considered based on the community detection. For example, the precise information delivery, e.g., Google AdWords [1] increases the transaction amounts for sending the advertisement information to the right person. Therefore, detecting communities is a popular research topic [2,3,4,5,6,7,8].

Many results focus on the disjoin community sets that each node belongs to exactly one community [2,3]. However, in the real-world networks, many people may belong to multiple communities, so the communities may overlap with each other. For example, an engineer may belong to many projects in a company. Thus, instead of strict partitions, fuzzy partitions are more appropriate for understanding the network structures [9,10]. Fuzzy partitions allow a node belongs to multiple communities simultaneously. Considering a real-world situation, some staff work together in a building, and the manager would like to track the movement history for each staff [11]. Each one may move to various rooms, and the move purpose comes from the role of each staff. When we treat the purpose of all staff to be the communities, the staff may belong to different communities.

The modularity function proposed by Newman and Girvan [12] is the famous measurement of network partitions to measure the structure of a given network. The modularity function calculates the difference between the number of real intra-community edges and the expected number of edges to identify the qualities of the communities. The partition with larger modularity value has better community structure than those with lower modularity values. Finding the partitions with maximum modularity is a straightforward solution to the community detection. However, the modularity maximization has been proved as an NP-hard problem [13], and finding the partition with maximum modularity is difficult. Therefore, many results are proposed to calculate the near optimal solutions, such as the random walk processes [14], the structural clustering [15], and the polynomial-time approximation algorithms [16].

**Figure 1:**
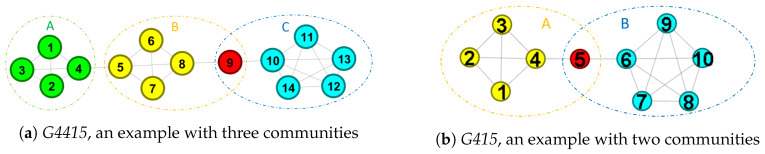
The benchmark with more than two communities and two communities.

On the other hand, besides the computation complexity, the modularity maximization has two problems in detecting communities:

**Resolution limits** Fortunato et al. introduced that small communities cannot be detected in large networks [17,18]. Since the null model of modularity provides the global connectivity, the expected number of edges between two small groups in a large network might be very small. Eventually, the two small groups will be treated as one community. Many approaches are proposed for solving resolution limits to provide high solution qualities, such as greedy algorithms [19,20], spectral algorithms [21,22,23], simulating annealing algorithms [24] and mathematical programing [25].
**Overlapping community** Some nodes may belong to several communities, so simply assigning the nodes to one community is difficult. Thus, the straightforward solution is to modify the modularity for allowing the nodes belonging to multiple communities at the same time [26,27,28,29,30]. Figure 1 shows two benchmarks about overlapping communities. In Figure 1a, the node $v_{9}$ is the overlapping node, and we assign $v_{9}$ to community *B* and *C*. Thus, we get three communities, and they are {{$v_{1}$, $v_{2}$, $v_{3}$, $v_{4}$}, {$v_{5}$, $v_{6}$, $v_{7}$, $v_{8}$, $v_{9}$}, {$v_{9}$, $v_{10}$, $v_{11}$, $v_{12}$, $v_{13}$, $v_{14}$}}. Moreover, $v_{5}$ is assigned to *A* and *B* in Figure 1b.

In this paper, we focus on the overlapping community detection, and propose the node weight allocation problem denoted by $NWA_{OCD}$ to formulate the community overlap. Since computing the partition with maximum modularity is NP-complete, decreasing the computation cost to seek the near optimal partitions is the popular approach in solving the overlapping community detection. The heuristic algorithms are outstanding in seeking better solutions in large search space, especially for the genetic algorithms (GAs) [2,3,8]. Therefore, some works consider GA as the core approach in their solutions. Mu et al. use a hybrid heuristic approach including GA and the simulated annealing to find out the communities [2]. Shang et al. use GA with an extra local search [3]. The heuristic algorithms perform well in seeking the solution with high quality in a large search space. However, the above results do not deal with the overlapping properties. The overlapping networks have various properties, so some approaches consider the multi-objective approach to find the balanced results [4,5,6,31]. The balanced results mean that most properties are considered, but the derived results may not be closed to the real-world properties. Therefore, Behera et al. check the similarity between each pair of nodes [8]. The node similarity is also considered by Ezeh et al. to the overlapping nodes and their neighbors [32]. To emphasize the community attribution of each node, Shakya et al. combine fuzzy with the GA to calculate the detail properties of the nodes [7]. Shakya et al. consider the GA to reduce the computation time without decreasing the solution quality too much and adopt the fuzzy communities to identify the overlapping nodes.

Even if some approaches provide the solutions with high modularity, the partitions may not reflect the properties of the real-world networks in some situations. We found that the solution quality could be refined by considering following issues: ignoring overlapping nodes, merging clusters, and reweighting nodes. Therefore, we consider the modularity to design the solution searcher of the approach $GA_{IMR}^{NWA}$. We firstly modify the fitness function in $GA_{IMR}^{NWA}$ to show the network properties by considering the null model, so the revised fitness function could output the partitions that are closer to the real-world behavior. Moreover, we design three refinement strategies to make the solutions to reflect the real-world properties.

In the simulation, we consider the synthetic network and popular networks that include Zachary Karate Club Network, Books about American Politics, and American College Football to evaluate the solution quality calculated by $GA_{IMR}^{NWA}$ and other approaches. The derived networks correctly reflect the real-world properties in the synthetic networks and the real-world networks. Moreover, the proposed refinement strategies are also evaluated, and the refinement strategies provide higher quality of the derived partitions in the perspective of the real-world behavior. Therefore, the simulation results show that $GA_{IMR}^{NWA}$ outputs the partitions, and the results are closed to the real-world properties.

This paper is organized as follows. The overlapping communities and the problem definition are introduced and formulated in Section 2. The proposed approach $GA_{IMR}^{NWA}$ is shown in Section 3, and the refinement strategies are also listed in this section. The simulation and comparisons are arranged in Section 4, and we show the network partitions in this section. Eventually, the conclusion and future works are stated in Section 5.

## 2. Preliminary

### 2.1. Modularity in Overlapping Communities

The community detection of a given network involves two processes. The first one is to find out the network structure and the other one is to determine the numbers of communities. Here we introduce the works proposed by Nepusz et al. [33] to explain the modularity in overlapping communities. Nepusz et al. consider a belonging coefficient matrix $U={\left[\alpha_{ic}\right]}_{n\times k}$, where *n* is the number of nodes, and *k* is a given number of communities. Each entry $\alpha_{ic}$ shows how strongly the node $v_{i}$ belongs to the community *c*. The constraint of the relationship between $v_{i}$ and all communities is:

(1) $$\sum_{c=1}^{k}\alpha_{ic}=1,\forall \alpha_{ic}\in \left[0,1\right],0<\sum_{i}^{n}\alpha_{ic}<n.$$

So, the objective function is:

(2) $$\begin{array}{c} D_{G}\left(U\right)=\sum_{i,j=1}^{n}w_{ij}{\left(\tilde{s_{ij}}-s_{ij}\right)}^{2}, \end{array}$$

where $w_{ij}$ is the predefined weight, $s_{ij}=\sum_{c=1}^{k}\alpha_{ic}\alpha_{jc}$, and $\tilde{s_{ij}}$ is the prior similarity of $v_{i}$ and $v_{j}$. By minimizing Equation (2), the nodes with high similarity will be grouped together. So, *U* with optimal result $D_{G}\left(U\right)$ is the overlapping community structure.

To determine an appropriate number of communities *k*, Nepusz et al. iteratively increase the value of *k* from 2, and then choose the value of *k* with the highest fuzzy modularity value calculated by Equation (3).

(3) $$\begin{array}{c} Q_{ov}^{Ne}=\frac{1}{2m}\sum_{c=1}^{k}\sum_{i,j=1}^{n}\left(A_{ij}-\frac{k_{i}k_{j}}{2m}\right)\alpha_{ic}\alpha_{jc} \end{array}$$

### 2.2. Problem Definition

The overlapping community detection problem is considered as a node weight allocation problem, denoted by $NWA_{OCD}$ for short. Given a network G(V,E), a maximum number of communities *k*, and a null model weight γ. Find a modified belonging coefficient matrix $M={\left[\lambda_{ic}\right]}_{n\times k}$, such that the $Q_{ov}^{{}^{\prime }}$ value is maximized. The objective function and constraints are:

(4) $$\begin{array}{cccc} \; & \max & \; & Q_{ov}^{{}^{\prime }}=\frac{1}{2m}\sum_{c=1}^{k}\sum_{i,j=1}^{n}\left(A_{ij}-\gamma \frac{k_{i}k_{j}}{2m}\right)\lambda_{ic}\lambda_{jc}\\ \; & s.t. & \; & \lambda_{ic}\in \left[0,1\right]\\ \; & \; & \; & \sum_{c=1}^{\left|C\right|}\lambda_{ic}^{inc_{f}}=1. \end{array}$$

We consider $inc_{f}$ as the increasing factor. Given $inc_{f}>1$, the total weight of an overlapping node over all communities is larger than one, i.e., $\sum_{c=1}^{k}\lambda_{ic}>1$. The total weight of a non-overlapping node is still equal to one exactly, i.e., $\sum_{c=1}^{k}\lambda_{ic}=1$.

By solving the $NWA_{OCD}$ problem, the overlapping community structure will be obtained by modifying the optimal solution. Note that if $inc_{f}=1$ and γ=1, Equation (4) is the same with Equation (5), which means the fuzzy modularity is a special case of the $NWA_{OCD}$ problem.

(5) $$\begin{array}{cccc} \; & \max & \; & Q_{ov}=\frac{1}{2m}\sum_{c=1}^{k}\sum_{i,j=1}^{n}\left(A_{ij}-\frac{k_{i}k_{j}}{2m}\right)\alpha_{ic}\alpha_{jc}\\ \; & s.t. & \; & \alpha_{ic}\in \left[0,1\right]\\ \; & \; & \; & \sum_{c}^{k}\alpha_{ic}=1. \end{array}$$

Although Griechisch et al. [34] apply the fuzzy modularity to find overlapping communities, there are still some networks are unresolved. We introduce the networks with more than two communities and two communities to show this issue. The benchmark is shown in Figure 1. The values of $Q_{ov}$ for G4415 and G415 are shown in Table 1. We can see that $v_{9}$ belongs to *B* in G4415 while $v_{5}$ belongs *A* in G415, and they are not overlapping nodes.

The major difference between Equations (4) and (5) is the coefficient matrix. Each entry in Equation (5) is unweighted while that is weighted in Equation (4). Therefore, we need a mapping as shown in the following equations.

(6) $$\begin{array}{cc} \; & \lambda_{ic}=\sqrt[{inc_{f}}]{\alpha_{ic}}\\ \; & \alpha_{ic}=\lambda_{ic}^{inc_{f}} \end{array}$$

## 3. Allocate Node Weight by Genetic Algorithms

Computing the partition with maximum modularity has been proved as the NP-complete problem [13]. Even if we consider the solution with high computation performance, e.g., the cloud computing [35,36] and the parallel computing [37], to compute the partitions for maximizing the modularity, it still requires huge computation resource. Therefore, we propose a GA-based approach to get the near-optimal solution with minimum computation. The proposed algorithm $GA_{IMR}^{NWA}$ includes two steps. We first apply GA to obtain a high-quality feasible solution, and then design three refinement strategies to improve the derived solution to modify the derived partition to be closer to the real-world behavior. In the following context, we will introduce the revised GA algorithm and the refinement strategies.

### 3.1. Genetic Algorithm

**Figure 2:**
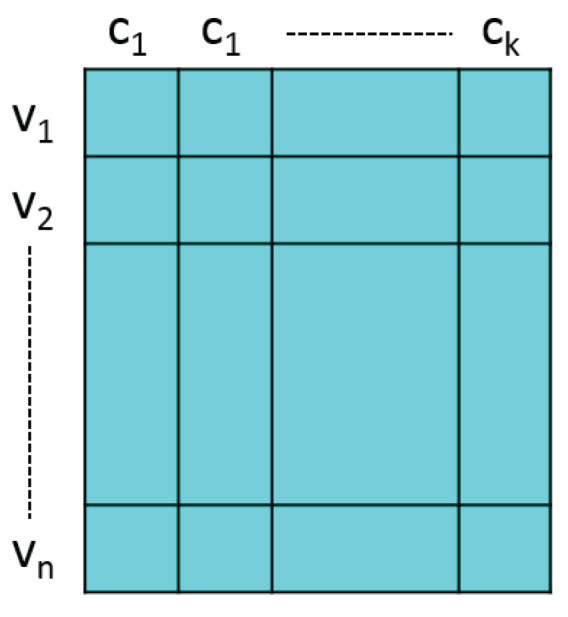
The representation of a chromosome.

The iterative process of GA as shown in Algorithm 1 includes three major processes: crossover, mutation, and selection. Before invoking the iterative process, the initial population *P* with $indi_{n}$ chromosomes will be determined firstly. Each chromosome is represented by $M={\left[\lambda_{ic}\right]}_{n\times k}$, as shown in Figure 2. Each entry $\lambda_{ic}$ is a weight to indicate the assignment from $v_{i}$ to *c*. The initial population is generated randomly, and each row of *M* must satisfy the problem constraints. Given a maximum number of iterations $max_{t}$, the GA then invokes following processes.

**Figure 3:**
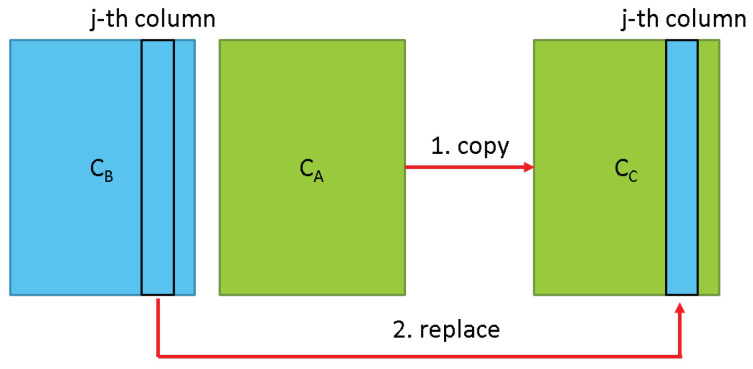
The idea of the crossover operation. Two chromosomes are switched the selected area to generate one offspring.

1. **Crossover**: we randomly select two chromosomes $C_{A}$ and $C_{B}$ form *P*, and a random column. The offspring is generated by the selected column of $C_{B}$ and the remaining part of $C_{A}$ as shown in Figure 3. The number of offsprings is determined by $indi_{n}$, and in other words, we will obtain $2\times indi_{n}$ chromosomes after the crossover.
2. **Mutation**: the mutation process is launched in 80% probability after finishing the crossover. Once the mutation is invoked, one $\lambda_{ic}$ of a randomly selected chromosome will be picked up within [−0.1,0.1]. Eventually, the offspring will be normalized to be a feasible solution to fit the requirements in $NWA_{OCD}$.
3. **Selection**: we consider the modularity to be the objective function, and finding the partition with maximum modularity is the purpose of GA. We use $Q_{ov}^{{}^{\prime }}$ to be the fitness function and calculate $Q_{ov}^{{}^{\prime }}$ of each solution. Moreover, all chromosomes are sorted in the descending order of $Q_{ov}^{{}^{\prime }}$. Computing the chromosomes with maximum $Q_{ov}^{{}^{\prime }}$ is the major goal of the GA, so we select top $indi_{n}$ individuals, and they will survive to the next generation.

**Algorithm 1:** Genetic algorithm for allocating node weight

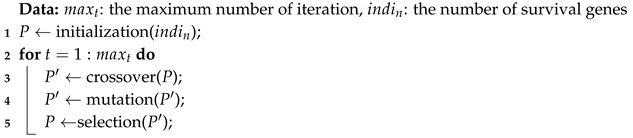



To keep the heavily overlapping nodes, a threshold $\alpha_{T}$ in terms of α is given. We transform $\alpha_{T}$ to the corresponding λ with the threshold $\lambda_{T}$ by Equation (6).

### 3.2. Refinement Strategies

GA provides an elite solution from the population, but this solution may not be suitable for all instances. In the pre-analysis phase, we observed three situations derived by $GA_{IMR}^{NWA}$, and we could receive better solutions by some extra processes. The situations are (1) lightly overlapping nodes, (2) mergeable clusters, and (3) reweight nodes. We call the processes that are used to get better solutions the “refinement strategies”. Therefore, we provide three refinement strategies to refine the solutions for the above situations, respectively.

**Ignore slight overlapping nodes** The overlapping degree of each λ is important for splitting the communities. Determining the community with low value of λ is easier than that with a higher value. We use a threshold $\lambda_{T}$ corresponding to Equation (6) to determine that the entry should be treated as an entry without overlaps. In addition, we also can derive $\lambda_{T}$ by Equation (6). When $\lambda <\lambda_{T}$, we set λ as zero. When $\lambda_{T}$ is set as a higher value, more entries will be assigned to single community.
**Merge clusters** Some small communities should be merged by other large community. If the overlapping ratio of any two communities is larger than a given merge threshold $m_{T}$, they should be simply merged to a single community. Given two non-empty communities, we define $ov_{ratio}=|C_{1}\cap C_{2}\left|/\min(\right|C_{1}\left|,\right|C_{2}\left|\right)$ to be the overlapping ratio. When $ov_{ratio}$ is larger than a given threshold, $C_{1}$ and $C_{2}$ will be merged.
**Reweight node values** To calculate the weight distribution of each overlapping node, directly converting λ to α via Equation (6) results in a situation that a node belongs to multiple communities but the majority of its weight is allocated to one community. To avoid this problem, we propose the reweight strategy. The weight should be proportional to the number of edges that $v_{i}$ linked in *c*. Moreover, if the neighbors of $v_{i}$ in *c* are more than the average number of nodes in *c*, *c* is more important than others for $v_{i}$. Given a community c, $avgNighbor_{c}=\sum_{i,j\in V(c)}A_{ij}/\left|V\left(c\right)\right|$ represents the average number of neighbors and $\alpha_{i}=\sum_{c\in C(i)}\sum_{j\in V(c)}A_{ij}/avgNighbor_{c}$ be the normalized term. Therefore, we have the new weight is:
  (7) $$\alpha_{ic}=\frac{1}{\alpha_{i}}\sum_{j\in V(c)}\frac{A_{ij}}{avgNighbor_{c}},$$
  where V(c) is the set of nodes belong to *c* and C(i) is the set of communities that $v_{i}$ belongs to. We use $\alpha_{i}$ for normalization, so we have $\sum_{c=1}^{k}\alpha_{ic}=1$.

## 4. Simulations

We consider a synthetic network and three real networks including Zachary Karate Club network, Books about American Politics, and American College Football to evaluate the performance of $GA_{IMR}^{NWA}$. The evaluation criteria involve detecting overlapping community structure, detecting meaningful communities, detecting dense overlaps, and detecting heavily overlapping nodes.

### 4.1. Synthetic Network

We consider *G210* as our synthetic network which has 210 nodes and four pre-defined communities *A*, *B*, *C* and *D*. Each of them has 60 nodes and 10 shared by any two continuous communities, i.e., $A=\{v_{1}:v_{60}\}$, $B=\{v_{51}:v_{110}\}$, $C=\{v_{101}:v_{160}\}$, and $D=\{v_{151}:v_{210}\}$. Note that *A* and *B* share nodes $\left\{v_{51},\ldots ,v_{60}\right\}$, *B* and *C* share nodes $\left\{v_{101},\ldots ,v_{110}\right\}$ and so on. Each pair of nodes has 3% chances to be linked to each other, and for each community they shared, an extra 55% chances for them to be linked. Thus, overlapping parts will be denser than non-overlapping parts [38].

**Figure 4:**
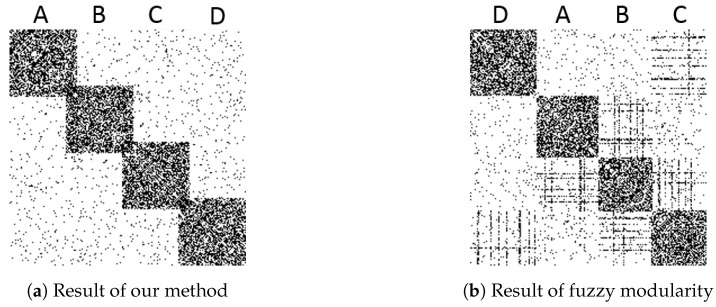
The comparison between $GA_{IMR}^{NWA}$ and fuzzy modularity.

Since the fuzzy modularity is a special case of the $NWA_{OCD}$ problem, we could use the same optimization strategy to solve the problem. The parameter settings are $inc_{f}=1.5$ and 1, $\alpha_{T}=0$, $m_{T}=-1$, k=6, and γ=1. Figure 4 shows the bitmaps of sorted adjacency matrices. The black and white points represent the entries of 1s and 0s respectively. The adjacency matrices are sorted by the following strategy:

1. Nodes are grouped by the detected community id. For the overlapping nodes, only the smallest id is counted.
2. For each *c*, all nodes are sorted in descending order of $\lambda_{ic}$. Therefore, the overlapping nodes will be in the bottom area of each community.

Figure 4a is the result obtained by $GA_{IMR}^{NWA}$. The dense blocks indicate four communities, and two continuous blocks have an overlapping part which is composed of overlapping nodes. In this result, all the overlapping and non-overlapping nodes are correctly identified. Figure 4b is the result of fuzzy modularity. Four communities are detected too, but no overlapping nodes are identified.

Although the maximum number of communities is six, only four communities were detected while the other two were empty communities. Since the number of communities could be captured by modularity [39], it is unnecessary to know the exactly value of number of communities in our method.

### 4.2. Zachary Karate Club Network

**Figure 5:**
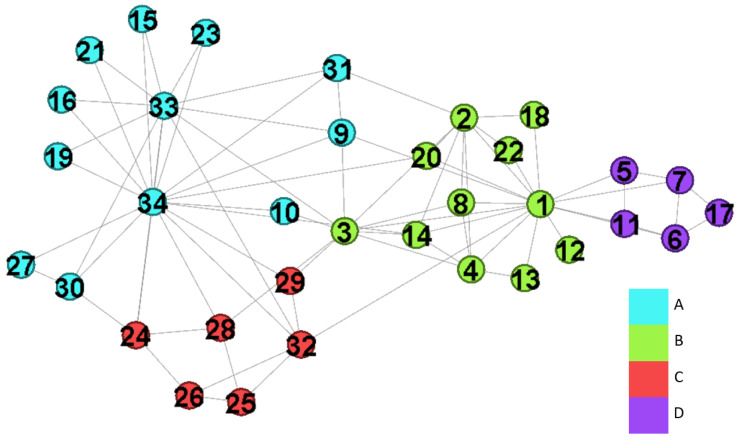
Detected communities of the karate network by fuzzy modularity.

Zachary karate club network [40] is a popular benchmark for community detection algorithms. It has 34 nodes and 78 edges while nodes are members and edges are friendships between them. This network includes two groups due to a disagreement between the administrator and the instructor. Figure 5 is the result captured by the fuzzy modularity. In this experiment, we evaluate the results with different $inc_{f}$ settings, and show the importance of “ignore slight overlapping nodes” and “reweight node values”. Finally, we apply our method on the case with the value k=2, and halved the null model.

#### 4.2.1. Effects of Weight Increasing Factor

We first evaluate the communities captured by $GA_{IMR}^{NWA}$ in the networks with $inc_{f}=\left\{1,1.2,1.5,1.7\right\}$ while $\alpha_{T}=0.01$, $m_{T}=-1$, k=8, and γ=1. The corresponding $Q_{ov}^{{}^{\prime }}=\left\{0.419,0.422,0.427,0.430\right\}$. We consider the fuzzy modularity with $inc_{f}=1$ as our baseline since it outputs the correct solution.

**Figure 6:**
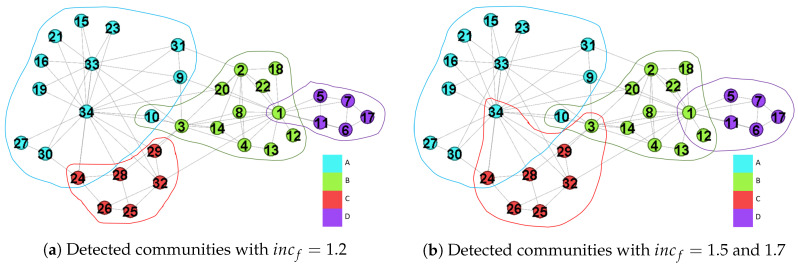
The communities detected by $GA_{IMR}^{NWA}$ under various $inc_{f}$ settings.

Figure 6a is the result with $inc_{f}=1.2$, and we get four communities and three overlapping nodes while λ is shown in Table 2a. The network separation in Figure 6a is identical to that in Figure 5, but maximizing the modularity outputs a larger one than that we derived. When $inc_{f}$ is increased from 1.2 to 1.5, we get two extra overlapping nodes, and they are $v_{12}$ and $v_{34}$. When $inc_{f}$ is set as 1.7, the values of λ are changes as shown in Table 2c, and others are identical to that derived by $inc_{f}=1.5$. Therefore, larger settings of $inc_{f}$ results in more overlapping nodes.

Considering that a node has only one edge connecting to an overlapping node, e.g., $v_{12}$, the isolation has the same property with that held by the overlapping node. Moreover, we found that $Q_{ov}^{{}^{\prime }}$ derived by $GA_{IMR}^{NWA}$ is higher than the optimal *Q*. It implies that the overlapping structure is easier to be detected as assigning higher weight to the overlapping nodes.

Here we consider an extreme case that all nodes are overlapped, i.e., $inc_{f}=4$. We analyze the obtained result, and then find the “duplicate communities”. Two or more communities are extremely overlapped with each other, and even some of them are just the same community.

**Figure 7:**
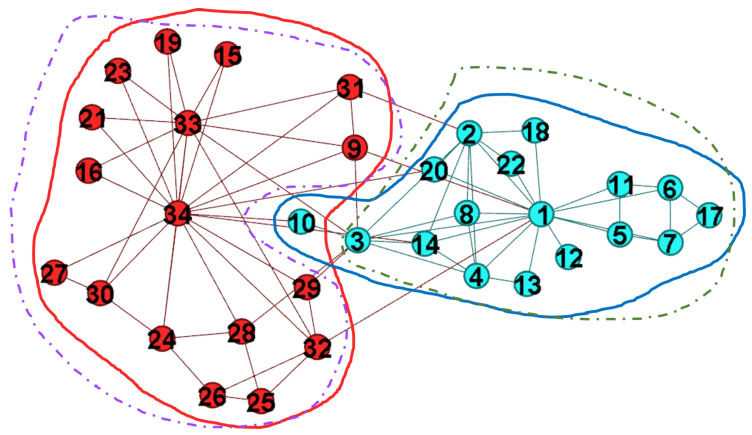
Duplicate communities result.

Figure 7 shows the fuzzy partition result. Four communities are detected, but two of them denoted by dotted lines are the subsets of the rest two communities denoted by solid lines. Therefore, two sets should be merged to a correct community. After merging the communities, we derive two communities, and there is only one overlapping node $v_{10}$. However, the value of $Q_{ov}^{{}^{\prime }}$ is decreased from 0.526 to 0.371 simultaneously.

Even if we derive the result with maximized value of $Q_{ov}^{{}^{\prime }}$, the solution does not show the correct properties of the communities. We use the refinement strategies to get the solution with lower quality but more closed to the real-world properties. Therefore, the refinement strategies are useful for improving the solution quality in terms of the real-world consideration.

#### 4.2.2. Effects of Ignoring Slight Overlapping Nodes

**Figure 8:**
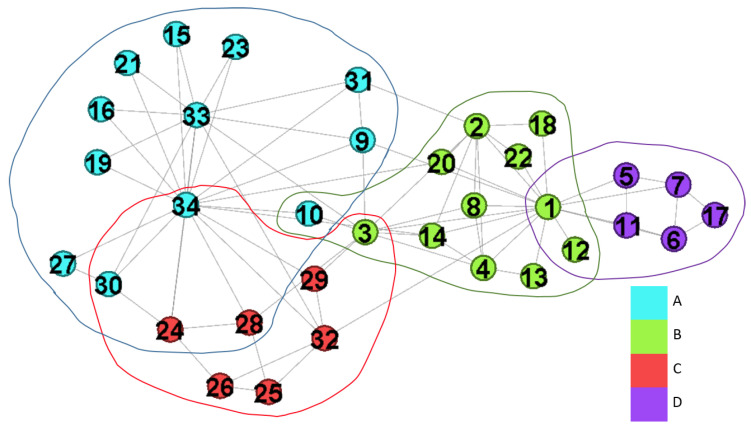
Detected communities with $inc_{f}=1.5$ (before ignoring).

We consider the network with $inc_{f}=1.5$ to evaluate the effects of the *ignore* step. The result with and without the *ignore* step are 0.427114 and 0.427117, respectively. Figure 8 and Table 3 are the detected communities and values of λ. Two overlapping nodes $v_{28}$ and $v_{30}$ are ignored. Since most of their weights were kept in a specific community, reducing the weights will not decrease $Q_{ov}^{{}^{\prime }}$ dramatically. Therefore, the process of ignoring slight overlapping nodes helps to keep those heavily overlapping nodes.

#### 4.2.3. Effects of Reweight Strategy

To emphasize the importance of the communities, we propose a reweight strategy to assign various weights. The result with reweight strategy is identical to that shown in Figure 6b. Table 4a,b show the value of λ without and with considering the reweight strategy, respectively. The reweight strategy reduces the gap of the number of edges for connecting the inside-community nodes and outside-community nodes. However, the structure of the main community may be changed after reweighting, because the values are inversely proportional to the average number of neighbors in the communities to that out of communities. For example, $v_{12}$ is unbalanced before reweighting, but the value of λ of $v_{12}$ reflect the real-world behavior.

#### 4.2.4. The Network with Two-Communities

**Figure 9:**
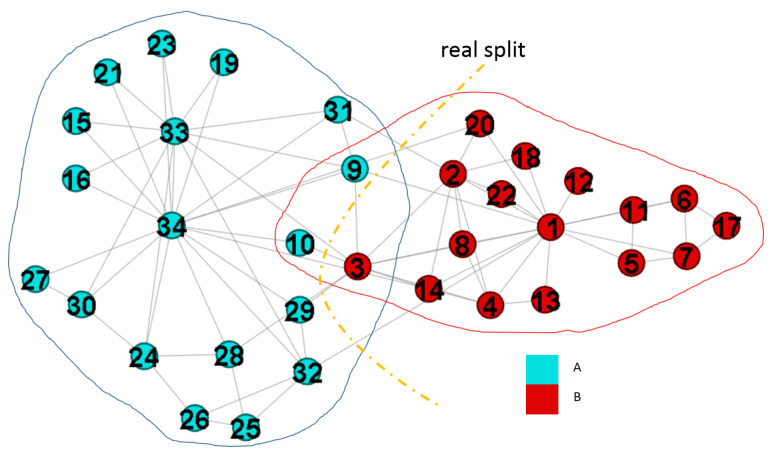
Detected communities with k=2, and γ=0.5.

We examine the network with exactly two communities to verify the property illustrated in Figure 1b can be captured by $GA_{IMR}^{NWA}$. We consider $inc_{f}=1.5$, $\alpha_{T}=0.01$, $m_{T}=-1$, k=2, and γ=0.5. In this case, we easily find out the overlapping nodes. The results are shown in Figure 9 and Table 5.

$GA_{IMR}^{NWA}$ derives three overlapping nodes as shown in Table 5. From Figure 9, we have $Q_{ov}^{{}^{\prime }}=0.628$, and the dotted curve is the real split of the club network. $v_{3}$ is the main overlapping node since it has a roughly balanced weight value. In summary, the two-community problem is solved by reducing the number of expected edges.

#### 4.2.5. Compare with Different Algorithms

In the above simulations, $GA_{IMR}^{NWA}$ detects two communities, and we compare the result with previous algorithms in this dataset. Shen et al. captured three overlapping communities [30], and the overlapping nodes are $v_{1}$, $v_{3}$ and $v_{9}$. However, $v_{12}$ is missed in the method of Shen et al. The property of the overlapping communities in $v_{12}$ is not discovered. The node $v_{12}$ has exactly one neighbor that is node $v_{1}$, so $v_{12}$ should have the same overlapping properties as that of $v_{1}$.

Chen et al. captured two overlapping communities [29], and their results are similar to ours as shown in Figure 9. Chen et al. found one overlapping node $v_{10}$. Node $v_{10}$ has two edges that one connects to the left community while the other one comments to the right community. Therefore, considering $v_{10}$ as the overlapping node is reasonable. However, the node $v_{3}$ has five edges where three edges connect to the left community while two connect to the right community. $v_{3}$ is more appropriate than $v_{10}$ to be the overlapping node.

From the above observation, the communities are split more precisely by $GA_{IMR}^{NWA}$ than the previous works. For the considerations of the split appropriateness, e.g., the number of detected communities, and the split correctness, e.g., the overlapping nodes, $GA_{IMR}^{NWA}$ provides more precise results than other approaches.

### 4.3. Books about American Politics

**Figure 10:**
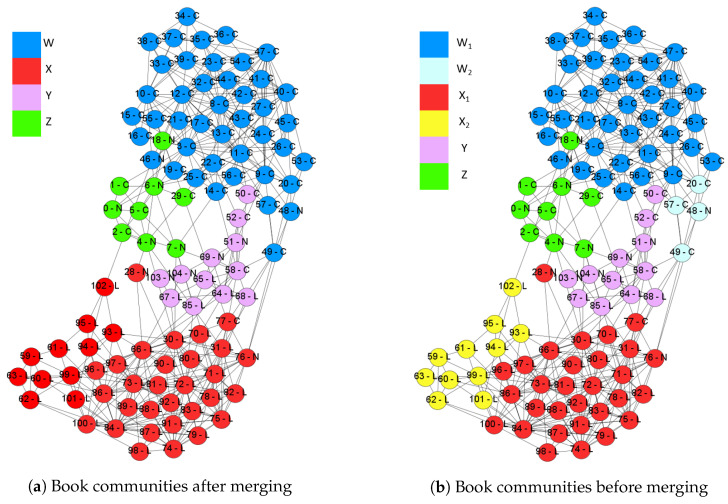
The book comparison between $GA_{IMR}^{NWA}$ with merging and without merging.

This network is built from the transaction data from amazon.com [41]. The network has 105 nodes and 441 edges while nodes indicate books and edges are frequent co-purchase events. The nodes are labeled by three categories including *liberal*, *neutral*, or *conservative*. Each category has 43, 13, and 49 nodes respectively. In this simulation, we consider $inc_{f}=1.5$, $\alpha_{T}=0.01$, $m_{T}=0.5$, k=8, and γ=1. We evaluate the performance of the merge strategy. Figure 10a,b are the solutions with and without merge strategies respectively. The text on each node is the node id and the real label. The results of $Q_{ov}^{{}^{\prime }}$ are 0.528 and 0.533 for the results with and without merge strategy.

#### 4.3.1. The Result with Merge Strategy

$GA_{IMR}^{NWA}$ with the merge strategy detects four communities denoted by *W*, *X*, *Y*, and *Z*. Most nodes belong to two large communities *W* and *X*, which are mainly consisted of *conservative* and *liberal* books respectively. Most *neutral* books belong to two small communities. This result is similar to that obtained by Newman [39]. Table 6 is the values of λ for ten overlapping nodes. There are four *neutral* nodes, that is 40% of all overlapping nodes and 30% of all *neutral* nodes. The result implies that *neutral* books are often co-purchased with different books.

#### 4.3.2. The Result without Merge Strategy

$GA_{IMR}^{NWA}$ without the merge strategy splits *W* and *X* into two parts respectively denoted by $W_{1}$, $W_{2}$, $X_{1}$ and $X_{2}$. A small community including $v_{48}$, $v_{49}$ and $v_{57}$ has been detected by the modularity maximization [25]. Therefore, we also found this community and labeled it by $W_{2}$.

Moreover, we also detect an extra community $X_{2}$. After analyzing the edge density of $X_{1}$ and $X_{2}$, they are both denser than the merged community *X*. Besides, the overlapped part is even denser as shown in Table 7. The density function definition is as follows:

(8) D(c)=1|V(c)|2∑i,j∈V(c)Aij2.

The overlapping ratios of ($W_{1}$, $W_{2}$) and ($X_{1}$, $X_{2}$) are 57% and 53%, respectively. High overlapping ratios indicate that we could merge each pair of them without decreasing $Q_{ov}^{{}^{\prime }}$ too much. Therefore, modularity can not detect $X_{2}$ because of high overlapping ratio and dense overlapped part. This result shows the dense overlaps can be discovered by $GA_{IMR}^{NWA}$ correctly.

### 4.4. American College Football

This is the network of American football games between Division IA colleges in 2000 [42]. It has 115 nodes, 613 edges and 12 conferences as shown in Table 8. Nodes are teams and edges are games between the corresponding two teams while nodes are labeled by the conferences they belong to. We apply $inc_{f}=1.5$, $\alpha_{T}=0.01$, $m_{T}=-1$, k=15, and γ=1 in this simulation.

**Figure 11:**
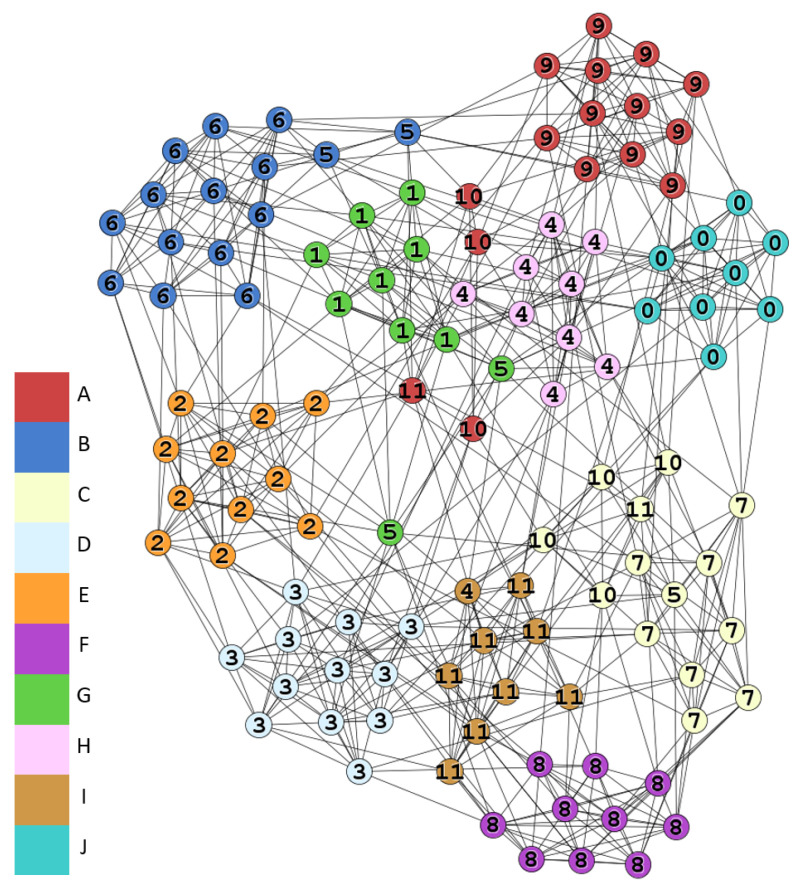
Football communities.

Figure 11 shows the result with $Q_{ov}^{{}^{\prime }}=0.607$, true labels are on the nodes. Ten communities and 17 overlapping nodes are detected. Most conferences are well matched to the detected communities except for the conferences *Independents* (Label 5) and *Sun Belt* (Label 10). There are total seven overlapping nodes in these two conferences. From Table 9, 41% overlapping nodes and 58% nodes are in the two conferences.

**Figure 12:**
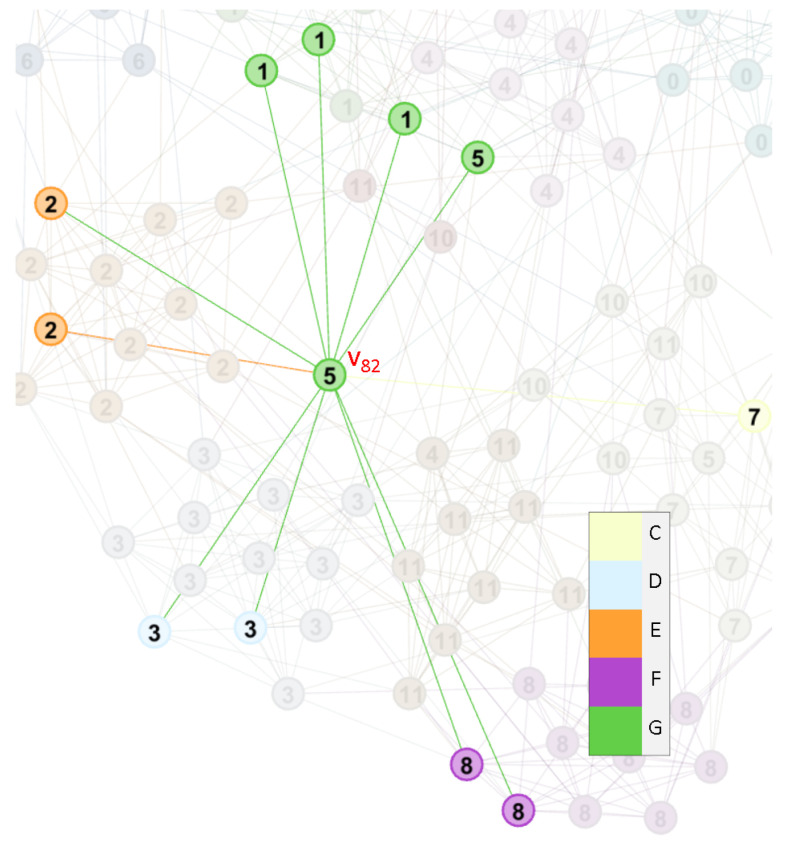
The node $v_{82}$ and its neighbors in football network.

The conference *Independents* has five teams, and only one game was held. This is the major reason that makes this conference undetectable. However, the teams often play with other teams in varied conferences, and this phenomenon results in the overlapping property. For example, $v_{82}$ is assigned to four communities, although it connected to community *G* with four edges. $v_{82}$ still connects to other three communities with a significant number of edges, so that is why it belongs to many communities simultaneously as shown in Figure 12. On the other hand, *Sun Belt* is in the similar situation. In this example, the heavily overlapping nodes could be detected by our method.

### 4.5. Dolphin Network

The Dolphin Network is a common benchmark for evaluating the overlapping communities. Some results consider the Dolphin Network to evaluate the community quality [26,43]. We compare the proposed $GA_{IMR}^{NWA}$ with related results in this simulation. The Dolphin Network includes 62 nodes and 159 edges, and two communities are detected eventually for a long-term observation.

**Figure 13:**
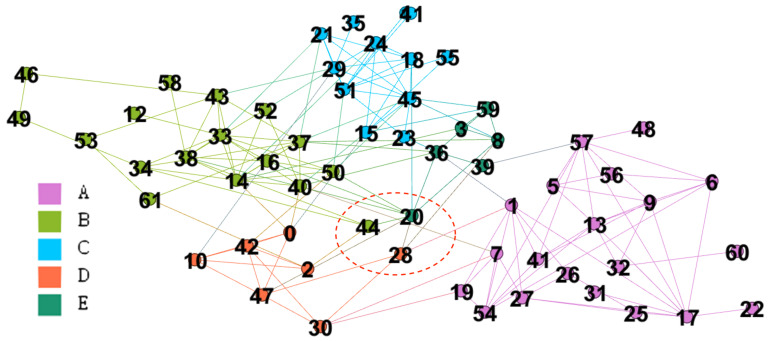
Five communities are detected by the proposed approach. There are three overlapping nodes when using $\lambda_{T}=0.9$. Therefore, the community B, C, D, and E could be merged by refinement strategy Ignore slight overlapping nodes, and we find two communities eventually.

The distribution of λ for overlapping nodes is listed in Table 10 while the separation with $Q^{\prime }=0.535$ is illustrated in Figure 13. According to the refinement strategy Ignore slight overlapping nodes, we get three overlapping nodes $v_{20},v_{28}$, and $v_{44}$ after decreasing the setting of $\lambda_{T}$ from 1.0 to 0.9. The overlapping nodes are marked by the red circle with dot lines, and they are marked by the overlapping nodes based on the distribution of λ. On the other hand, we also consider $m_{T}=-1$ in Dolphin network as the same setting in the above simulations. The community B, C, D, and E are merged according to the refinement strategy Merge clusters. Eventually, we get two communities.

Nicosia et al. found four communities in Dolphin network [26]. The overlapping nodes are mentioned, but the authors did not list the overlapping nodes. Wang and Fleury provided detail analysis and found two communities from Dolphin network with Q=0.385[43]. The separation is acceptable, but the network structure is not so strong comparing to Figure 13. After considering the refinement strategies, the separation derived by the proposed $GA_{IMR}^{NWA}$ is similar to that provided by Wang and Fleury in [43], but the structure of our network is stronger than the network in [43]. In summary, the refinement strategies are useful in revising the network separation to be closer to the real-world behavior, and the strength of the network structure is also improved.

## 5. Conclusion and Discussion

Given a network, the modularity is used for measuring the partition quality while the fuzzy clustering recognizes the overlapping communities. Combining above concepts together to be the fuzzy modularity is an appropriate method to formulate the structure of the given network with overlapping communities. Maximizing the modularity outputs the partition with well network structure, but computing the partition with maximum modularity requires huge computation cost. Therefore, the heuristic algorithms are outstanding in seeking high quality solution from a large search space, and we can find some research results of using heuristic algorithms for finding the partitions with maximum modularity. However, there are some special cases that we have to deal with. We find out three common situations from the partitions derived from the GA with modularity maximization and propose three solution refinement strategies to ignore overlapping nodes, merge clusters, and reweight nodes to separate the network to be closer the real-world behaviors. Moreover, we modify the fitness function of the GA to consider the null model for measuring the distance between the derived partition and the random graph. Thus, the simulation results show that the proposed $GA_{IMR}^{NWA}$ provide significant improvement comparing with previous approaches. The derived partition may not always have maximum modularity, but the community structure is more reasonable than the partitions derived by previous works. $GA_{IMR}^{NWA}$ measures the connectivity of nodes and reweight the overlapping nodes to reflect the correct properties in the given networks. Eventually, $GA_{IMR}^{NWA}$ determines the partitions appropriately, but the heavily overlapping nodes may be marked as the interior nodes by other approaches.

**Figure 14:**
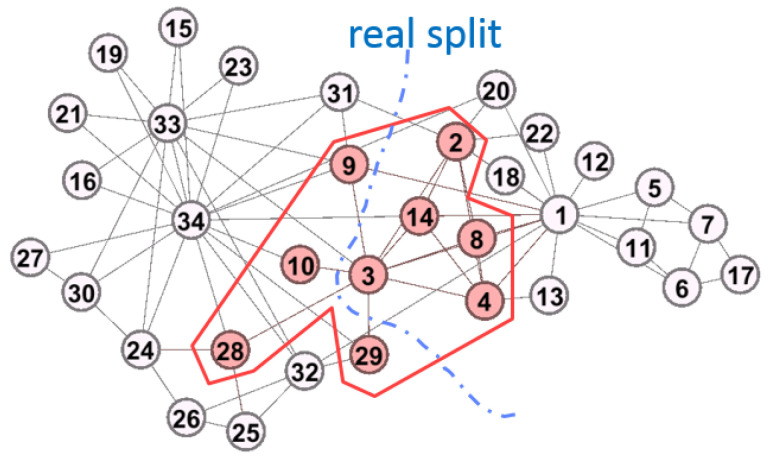
The 5th detected community of the karate network.

The overlapping nodes could be detected and provided appropriate allocation by $GA_{IMR}^{NWA}$. During the simulations, we found some extension works that will be address in the future, and they are listed as follows:

1. In our simulations, we got an interesting result as shown in Figure 14 from the karate network with $inc_{f}=2$. The result consists of three communities, and they are grouped by $v_{33}$, $v_{3}$ and $v_{1}$. The community with $v_{3}$ that the nodes are marked by red could be consider as an overlapping set. It means that the networks not only have overlapping nodes but also overlapping groups. Thus, applying the fuzzy concept to the communities will eliminate the group with $v_{3}$, and they may be more closed to the real-world behavior. Since the members in the group with $v_{3}$ may belong to different communities based on the situations, e.g., the competitions or the events. Therefore, assigning the red nodes to any community may be inappropriate.
2. The proposed algorithm invokes GA to compute the preliminary partitions and then adopts proposed refinement strategies to correct the partitions by the secondary processes. The refinement strategies could be considered as the local search to improve the partition quality in each iteration. However, it is a tradeoff between the computation cost and the partition quality. Once the refinement strategies are modified from the external processes to the internal processes in GA, the computation cost will be increased. Moreover, the given networks may not always consist of the target properties that could be improved by the refinement strategies. Therefore, the refinement strategies could be designed as local search approaches, but the trigger of launching the local search approaches should be analyzed in the future.

## Figures and Tables

**Table 1:** The values of $Q_{ov}$ in G4415 and G415.

(**a**) The $Q_{ov}$ values with different assignments of $v_{9}$ in G4415.			
$\alpha_{9,B}$	$\alpha_{9,C}$	$\alpha_{9,D}$	$Q_{ov}$
1	0	0	0.5736
0.7	0.3	0	0.5709
0.3	0.7	0	0.5664
0	1	0	0.5624
0	0	1	0.5560
(**b**) The $Q_{ov}$ values with different assignments of $v_{5}$ in G415.			
$\alpha_{5,A}$	$\alpha_{5,B}$	$\alpha_{5,C}$	$Q_{ov}$
1	0	0	0.4305
0	0	1	0.4151
0	1	0	0.4058

**Table 2:** The comparison with various $inc_{f}$ settings.

(**a**) λ values of overlapping nodes in Figure 6a with $inc_{f}=1.2$				
Node	$\lambda_{iA}$	$\lambda_{iB}$	$\lambda_{iC}$	$\lambda_{iD}$
$v_{1}$		0.967		0.068
$v_{10}$	0.747	0.362		
$v_{24}$	0.419		0.696	
(**b**) λ values of overlapping nodes in Figure 6b with $inc_{f}=1.5$				
Node	$\lambda_{iA}$	$\lambda_{iB}$	$\lambda_{iC}$	$\lambda_{iD}$
$v_{1}$		0.917		0.246
$v_{3}$		0.986	0.075	
$v_{10}$	0.700	0.556		
$v_{12}$		0.993		0.048
$v_{24}$	0.553		0.703	
$v_{34}$	0.993		0.048	
(**c**) λ values of overlapping nodes in Figure 6b with $inc_{f}=1.7$				
Node	$\lambda_{iA}$	$\lambda_{iB}$	$\lambda_{iC}$	$\lambda_{iD}$
$v_{1}$		0.888		0.369
$v_{3}$		0.987	0.108	
$v_{10}$	0.694	0.636		
$v_{12}$		0.926		0.290
$v_{24}$	0.600		0.726	
$v_{34}$	0.989		0.097	

**Table 3:** λ values of overlapping nodes in Figure 8 with $inc_{f}=1.5$ (before ignoring).

Node	$\lambda_{\mathrm{iA}}$	$\lambda_{\mathrm{iB}}$	$\lambda_{\mathrm{iC}}$	$\lambda_{\mathrm{iD}}$
$v_{1}$		0.917		0.246
$v_{3}$		0.986	0.075	
$v_{10}$	0.700	0.556		
$v_{12}$		0.993		0.048
$v_{24}$	0.553		0.703	
$v_{28}$	0.002		0.999	
$v_{30}$	0.999		0.004	
$v_{34}$	0.993		0.048	

**Table 4:** The comparison of $GA_{IMR}^{NWA}$ with reweighting and without reweighting.

(**a**) Before reweighting				
Node	$\lambda_{iA}$	$\lambda_{iB}$	$\lambda_{iC}$	$\lambda_{iD}$
$v_{1}$		0.917		0.246
$v_{3}$		0.986	0.075	
$v_{10}$	0.700	0.556		
$v_{12}$		0.993		0.048
$v_{24}$	0.553		0.703	
$v_{34}$	0.993		0.048	
(**b**) After reweighting				
Node	$\alpha_{iA}$	$\alpha_{iB}$	$\alpha_{iC}$	$\alpha_{iD}$
$v_{1}$		0.611		0.389
$v_{3}$		0.709	0.291	
$v_{10}$	0.52	0.48		
$v_{12}$		0.440		0.560
$v_{24}$	0.468		0.532	
$v_{34}$	0.725		0.275	

**Table 5:** λ values of overlapping nodes in Figure 9.

Node	$\lambda_{\mathrm{iA}}$	$\lambda_{\mathrm{iB}}$
$v_{3}$	0.493	0.753
$v_{9}$	0.987	0.071
$v_{10}$	0.984	0.085

**Table 6:** λ values of overlapping nodes in Figure 10a.

Node	$\lambda_{\mathrm{iW}}$	$\lambda_{\mathrm{iX}}$	$\lambda_{\mathrm{iY}}$	$\lambda_{\mathrm{iZ}}$	Label
$v_{3}$	0.986			0.076	conservative
$v_{7}$			0.254	0.913	neutral
$v_{9}$	0.975		0.11		conservative
$v_{18}$	0.528			0.724	neutral
$v_{22}$	0.955			0.164	conservative
$v_{25}$	0.922			0.236	conservative
$v_{28}$		0.72		0.533	neutral
$v_{46}$	0.921	0.238			neutral
$v_{50}$	0.458		0.781		conservative
$v_{85}$			0.981	0.093	liberal

**Table 7:** Density value of each part of community *X*.

	*X*	$X_{1}$	$X_{2}$	$X_{1}\cap X_{2}$
D(c)	0.20	0.27	0.34	0.63

**Table 8:** Labels of conferences.

Label	Conference	#Teams	Label	Conference	#Teams
**0**	Atlantic Coast	9	**6**	Mid-American	13
**1**	Big East	8	**7**	Mountain West	8
**2**	Big Ten	11	**8**	Pacific Ten	10
**3**	Big Twelve	12	**9**	Southeastern	12
**4**	Conference USA	10	**10**	Sun Belt	7
**5**	Independents	5	**11**	Western Athletic	10

**Table 9:** λ values of overlapping nodes in Figure 11.

Node	$\lambda_{\mathrm{iA}}$	$\lambda_{\mathrm{iB}}$	$\lambda_{\mathrm{iC}}$	$\lambda_{\mathrm{iD}}$	$\lambda_{\mathrm{iE}}$	$\lambda_{\mathrm{iF}}$	$\lambda_{\mathrm{iG}}$	$\lambda_{\mathrm{iH}}$	$\lambda_{\mathrm{iI}}$	$\lambda_{\mathrm{iJ}}$	Label
$v_{2}$				0.06	0.99						**2**
$v_{8}$			0.058			0.991					**8**
$v_{9}$			0.971			0.123					**7**
$v_{11}$			0.937	0.204							**10**
$v_{23}$			0.981			0.094					**7**
$v_{36}$	0.575	0.682									**5**
$v_{44}$							0.11	0.975			**4**
$v_{50}$			0.902			0.274					**10**
$v_{58}$	0.961								0.149		**11**
$v_{66}$	0.067							0.989			**4**
$v_{67}$			0.05						0.993		**11**
$v_{69}$			0.992						0.054		**10**
$v_{78}$			0.05			0.992					**8**
$v_{80}$							0.941	0.121		0.126	**5**
$v_{82}$				0.065	0.082	0.097	0.953				**5**
$v_{97}$	0.704			0.326				0.368			**10**
$v_{112}$	0.065							0.989			**4**

**Table 10:** λ values of overlapping nodes in Figure 13.

Node	$\lambda_{\mathrm{iA}}$	$\lambda_{\mathrm{iB}}$	$\lambda_{\mathrm{iC}}$	$\lambda_{\mathrm{iD}}$	$\lambda_{\mathrm{iE}}$
$v_{0}$		0.008		0.999	
$v_{1}$	0.998				0.023
$v_{2}$		0.076		0.986	
$v_{7}$	0.990			0.061	
$v_{8}$				0.022	0.998
$v_{15}$			0.999	0.000	0.013
$v_{19}$	0.986			0.074	
$v_{20}$		0.361		0.364	0.682
$v_{23}$			0.909		0.261
$v_{28}$				0.823	0.400
$v_{30}$	0.051			0.992	
$v_{36}$		0.013			0.999
$v_{37}$		0.990			0.062
$v_{39}$	0.255				0.912
$v_{40}$		0.998			0.021
$v_{44}$		0.844		0.362	0.038
$v_{45}$			0.992		0.053
$v_{47}$				0.999	0.011
$v_{50}$		0.928	0.175	0.103	
$v_{52}$		0.999	0.009		
$v_{59}$			0.138		0.965
$v_{61}$		0.925		0.229	

## Abbreviations

The following abbreviations are used in this manuscript:

GA	Genetic Algorithms

## References

[1] Rosso M.A., McClelland M.K., Jansen B.J., Fleming S.W. (2019). Using Google AdWords in the MBA MIS course. J. Inf. Syst. Educ..

[2] Mu C.H., Xie J., Liu Y., Chen F., Liu Y., Jiao L.C. (2015). Memetic algorithm with simulated annealing strategy and tightness greedy optimization for community detection in networks. Appl. Soft Comput..

[3] Shang R., Bai J., Jiao L., Jin C. (2013). Community detection based on modularity and an improved genetic algorithm. Physica A.

[4] Bello-Orgaz G., Salcedo-Sanz S., Camacho D. (2018). A multi-objective genetic algorithm for overlapping community detection based on edge encoding. Inf. Sci..

[5] Li Z., Liu J. (2016). A multi-agent genetic algorithm for community detection in complex networks. Physica A.

[6] Yuxin Z., Shenghong L., Feng J. (2017). Overlapping community detection in complex networks using multi-objective evolutionary algorithm. Comput. Appl. Math..

[7] Shakya H.K., Singh K., Biswas B. An efficient genetic algorithm for fuzzy community detection in social network. Proceedings of the International Conference on Advanced Informatics for Computing Research.

[8] Behera R.K., Naik D., Rath S.K., Dharavath R. (2020). Genetic algorithm-based community detection in large-scale social networks. Neural Comput. Appl..

[9] Binesh N., Rezghi M. (2018). Fuzzy clustering in community detection based on nonnegative matrix factorization with two novel evaluation criteria. Appl. Soft Comput..

[10] Naderipour M., Zarandi M.H.F., Bastani S. (2020). A type-2 fuzzy community detection model in large-scale social networks considering two-layer graphs. Eng. Appl. Artif. Intell..

[11] Yang C.T., Chen S.T., Den W., Wang Y.T., Kristiani E. (2019). Implementation of an intelligent indoor environmental monitoring and management system in cloud. Future Generat. Comput. Syst..

[12] Newman M.E.J., Girvan M. (2004). Finding and evaluating community structure in networks. Phys. Rev. E.

[13] Brandes U., Delling D., Gaertler M., Goerke R., Hoefer M., Nikoloski Z., Wagner D. (2006). Maximizing Modularity is hard. arXiv.

[14] Lai D., Lu H., Nardini C. (2010). Enhanced modularity-based community detection by random walk network preprocessing. Phys. Rev. E.

[15] Huang J., Sun H., Han J., Deng H., Sun Y., Liu Y. SHRINK: A Structural Clustering Algorithm for Detecting Hierarchical Communities in Networks. Proceedings of the 19th Conference on Information and Knowledge Management.

[16] Dinh T.N., Thai M.T. (2013). Community detection in scale-free networks: Approximation algorithms for maximizing modularity. IEEE J. Select. Areas Commun..

[17] Fortunato S., Barthélemy M. (2007). Resolution limit in community detection. Proc. Natl. Acad. Sci. USA.

[18] Arenas A., Fernández A., Gómez S. (2008). Analysis of the structure of complex networks at different resolution levels. New J. Phys..

[19] Newman M.E.J. (2004). Fast algorithm for detecting community structure in networks. Phys. Rev. E.

[20] Clauset A., Newman M.E.J. (2004). Moore, C. Finding community structure in very large networks. Phys. Rev. E.

[21] Newman M.E.J. (2006). Finding community structure in networks using the eigenvectors of matrices. Phys. Rev. E.

[22] White S., Smyth P. A spectral clustering approach to finding communities in graph. Proceedings of the SIAM International Conference on Data Mining.

[23] Richardson T., Mucha P.J., Porter M.A. (2009). Spectral Tripartitioning of Networks. Phys. Rev. E.

[24] Guimera R., Amaral L.A.N. (2005). Functional cartography of complex metabolic networks. Nature.

[25] Agarwal G., Kempe D. (2008). Modularity-maximizing graph communities via mathematical programming. EPJB.

[26] Nicosia V., Mangioni G., Carchiolo V., Malgeri M. (2009). Extending the definition of modularity to directed graphs with overlapping communities. J. Stat. Mech.

[27] Reichardt J., Bornholdt S. (2006). Statistical mechanics of community detection. Phys. Rev. E.

[28] Liu J. (2010). Fuzzy modularity and fuzzy community structure in networks. Eur. Phys. J. B.

[29] Chen D., Shang M., Lv Z., Fu Y. (2010). Detecting overlapping communities of weighted networks via a local algorithm. Physica A.

[30] Shen H.-W., Cheng X.-Q., Guo J.-F. (2009). Quantifying and identifying the overlapping community structure in networks. J. Stat. Mech..

[31] Choong J.J., Liu X., Murata T. (2020). Optimizing Variational Graph Autoencoder for Community Detection with Dual Optimization. Entropy.

[32] Ezeh C., Tao R., Zhe L., Yiqun W., Ying Q. (2019). Multi-Type Node Detection in Network Communities. Entropy.

[33] Nepusz T., Petróczi A., Nógyessy L., Bazsó F. (2008). Fuzzy communities and the concept of bridgeness in complex networks. Phys. Rev. E.

[34] Griechisch E., Pluhár A. (2011). Community detection by using the extended modularity. Acta Cybern..

[35] Yang C.T., Shih W.C., Chen L.T., Kuo C.T., Jiang F.C., Leu F.Y. (2015). Accessing medical image file with co-allocation HDFS in cloud. Future Generat. Comput. Syst..

[36] Yang C.T., Liu J.C., Chen S.T., Lu H.W. (2017). Implementation of a big data accessing and processing platform for medical records in cloud. J. Med. Syst..

[37] Natarajan S., Vairavasundaram S., Ravi L. (2019). Optimized fuzzy-based group recommendation with parallel computation. J. Intell. Fuzzy Syst..

[38] Yang J., Leskovec J. Overlapping Community Detection at Scale: A Nonnegative Matrix Factorization Approach. Proceedings of the Sixth ACM International Conference on Web Search and Data Mining.

[39] Newman M.E.J. (2006). Modularity and community structure in networks. Proc. Natl. Acad. Sci. USA.

[40] Zachary W.W. (1977). An information flow model for conflict and fission in small groups. J. Anthropolog. Res..

[41] Krebs V. http://www.orgnet.com/.

[42] Girvan M., Newman M.E.J. (2002). Community structure in social and biological networks. Proc. Natl. Acad. Sci. USA.

[43] Wang Q., Fleury E. (2011). Uncovering overlapping community structure. Complex Networks.

